# The prognostic impact of perioperative dynamic changes in cachexia index in patients with hepatocellular carcinoma

**DOI:** 10.1002/ags3.12804

**Published:** 2024-04-16

**Authors:** Munetoshi Akaoka, Koichiro Haruki, Yuto Yamahata, Kohei Okazaki, Kenei Furukawa, Masashi Tsunematsu, Yoshihiro Shirai, Shinji Onda, Michinori Matsumoto, Toru Ikegami

**Affiliations:** ^1^ Division of Hepatobiliary and Pancreatic Surgery, Department of Surgery The Jikei University School of Medicine Tokyo Japan

**Keywords:** biomarker, cachexia, dynamic change, hepatectomy, hepatocellular carcinoma

## Abstract

**Background:**

The cachexia index (CXI), which consists of skeletal muscle, inflammation, and nutritional status, has been associated with prognosis in patients with hepatocellular carcinoma (HCC). We hypothesized that dynamic changes in CXI might be associated with long‐term outcomes in HCC.

**Methods:**

This study comprised 131 patients who had undergone primary hepatic resection for HCC between 2008 and 2019. Preoperative CXI (pre‐CXI) and postoperative CXI (post‐CXI) were calculated by the following formula: skeletal muscle index x serum albumin level / neutrophil‐to‐lymphocyte ratio. Pre‐ and post‐CXI were classified into two groups (high vs. low). We retrospectively investigated the association of perioperative dynamic changes in CXI with disease‐free and overall survival.

**Results:**

In multivariate analyses, negative HBs‐antigen (*p* = 0.02), high serum PIVKA‐II level (*p* < 0.01), poor tumor differentiation (*p* = 0.02), multiple tumors (*p* < 0.01), microvascular invasion (*p* < 0.01), partial resection (*p* < 0.01), postoperative complications (*p* < 0.01), and low‐pre‐CXI (*p* < 0.01) were significant predictors of disease‐free survival, while high ICG_R15_ (*p* = 0.01), poor tumor differentiation (*p* < 0.01), multiple tumors (*p* = 0.01), postoperative complications (*p* < 0.01), low‐pre‐CXI (*p* < 0.01), and low‐post‐CXI (*p* < 0.01) were significant predictors of overall survival. Low‐post‐CXI was associated with older age (*p* = 0.045), larger tumor (*p* < 0.01), longer operation time (*p* = 0.047), greater intraoperative bleeding (*p* < 0.01), and intraoperative blood transfusion (*p* < 0.01). Moreover, dynamic changes in CXI were associated with overall survival in each subgroup of patients with low‐pre‐CXI (*p* = 0.02) or high‐pre‐CXI (*p* = 0.03).

**Conclusions:**

Not only post‐CXI but also dynamic changes in CXI from pre‐ to post‐hepatectomy can be a prognostic indicator of HCC, providing a compelling rationale for aggressive perioperative nutritional and physical interventions to improve long‐term outcomes.

## Graphical abstract

Preoperative cachexia index (CXI), which consists of skeletal muscle, inflammation, and nutritional status, has been associated with prognosis in patients with hepatocellular carcinoma (HCC). We found that postoperative CXI and dynamic changes in CXI from before to after hepatic resection were significantly associated with overall survival in patients with HCC. Interestingly, intraoperative blood loss in the preoperative high CXI group and age in the preoperative low CXI group were associated with low postoperative CXI.

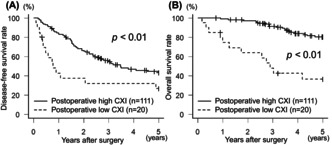

## 1 INTRODUCTION

Hepatocellular carcinoma (HCC) is the seventh most common cancer and the second leading cause of cancer mortality worldwide, accounting for 75%–85% of primary liver cancers.^1^ The general curative treatment for HCC is surgical resection, but the recurrence rate after hepatic resection remains high,^2^ suggesting the need to improve the therapeutic strategy for HCC. The risk stratification is important to develop more personalized therapy, which may improve the prognosis of HCC after resection. It has been reported that pre‐ and postoperative nutritional and inflammatory indexes reflect the prognosis after surgery for HCC.^3^

Cachexia is a complex wasting syndrome seen in patients with cancer and many chronic diseases.^4^ Cancer cachexia, characterized by malnutrition, systemic inflammatory response, and loss of skeletal muscle mass, has been associated with poor prognosis in patients with cancer, including HCC.^5^ The degree of cancer cachexia can be represented by the cachexia index (CXI), which consists of skeletal muscle, inflammation, and nutritional status.^6^, ^7^, ^8^ Although each component that contributes to forming cancer cachexia has been associated with prognosis, CXI, a comprehensive biomarker of cancer cachexia, has been more strongly associated with poor oncological outcomes in patients with HCC.^9^ Given major surgeries including hepatectomy can develop postoperative malnutrition, inflammation, and loss of skeletal muscle mass due to their invasiveness,^10^, ^11^ the degree of cachexia might change from before to after surgery. However, the prognostic association of postoperative CXI or dynamic changes in CXI with survival in patients with cancer including HCC has not been investigated. Therefore, in this study, we retrospectively investigated whether postoperative CXI and dynamic changes in CXI from pre‐ to post‐hepatectomy reflect the prognosis of patients after primary hepatic resection for HCC.

## 2 MATERIALS AND METHODS

### 2.1 Patient selection

This retrospective study analyzed 131 patients with HCC who underwent primary hepatic resection at the Department of Surgery, Jikei University Hospital, Tokyo, Japan, between January 2008 and December 2019. We excluded patients with other malignancies, perioperative mortality, and unavailable data on preoperative and postoperative CXI. Clinical information, surgical and pathological findings, and postoperative clinical courses were collected from medical records and a prospectively maintained database of patients at our institution. This study was approved by the Ethics Committee of the Jikei University School of Medicine (#27–177).

### 2.2 Treatment and follow‐up

Generally, the extent of hepatic resection was determined based on the retention rate of indocyanine green (ICG) at 15 min (ICG_R15_) and the hepatic reserve, as described by Miyagawa et al.^12^ The nomenclature of the liver segments and types of operation followed the Tokyo 2020 terminology.^13^ The type of resection was either anatomical resection (hemihepatectomy, sectionectomy, or segmentectomy) or non‐anatomical limited partial resection.^14^ The Tumor‐Nodes‐Metastasis (TNM) classification was based on the tumor pathology and the General Rules for the Clinical and Pathological Study of Primary Liver Cancer by the Liver Cancer Study Group of Japan.^15^

The recurrence of HCC was defined as newly detected hypervascular hepatic or extrahepatic tumors by ultrasonography, computed tomography, magnetic resonance imaging, or angiography with or without an increase in the serum alpha‐fetoprotein (AFP) levels or levels of protein induced by vitamin K absence or antagonist‐II (PIVKA‐II). For recurrent HCC in the liver, repeated hepatic resection, local ablation therapy, or transcatheter arterial chemoembolization (TACE) was performed based on the hepatic functional reserve, judged mainly by ICG_R15_. Extrahepatic recurrences were mainly treated with molecular‐targeted drugs. Postoperative complications were classified according to the Clavien–Dindo classification.^16^

### 2.3 Assessment of the cachexia index (CXI)

The skeletal muscle index (SMI) was calculated using the right psoas muscle based on a previously described method.^9^ We simply measured the lengths of the major and minor axes of the right psoas muscle at the caudal end of the third lumbar vertebra, and calculated the psoas muscle mass area (PMA). SMI was calculated as PMA / the height squared (cm^2^/m^2^). We assessed preoperative PMA by computed tomography (CT) taken last before surgery and postoperative PMA by CT taken closest to 90 days between 45 and 135 days after surgery as the first postoperative surveillance is performed at 3 months after surgery. The height was measured preoperatively. As previously described,^17^ the neutrophil‐to‐lymphocyte ratio (NLR) was calculated by dividing the absolute neutrophil count by the absolute lymphocyte count. We assessed preoperative NLR by the complete blood count within 7 days before surgery and postoperative NLR by the complete blood count performed on the same day as the CT scan date used to calculate the postoperative psoas muscle. We then calculated CXI as SMI × serum albumin level (g/L) / NLR.^6^, ^7^, ^8^, ^9^ Given skeletal muscle mass varies by sex, we classified preoperative CXI into two groups (high vs. low) based on the receiver operating characteristic (ROC) curves in strata of sex. Preoperative CXI cut‐off level for each sex was determined by maximizing the Youden index (the sum of sensitivity and specificity) for predicting 5‐year survival on each ROC curve. The cut‐off value for postoperative CXI was set to the same value as for preoperative CXI.

### 2.4 Definition of sarcopenia

We assessed sarcopenia using preoperative PMA. As previously described,^18^ preoperative PMA below the median for each sex was defined as sarcopenia. The sex‐specific cut‐off values for preoperative PMA were 35.1 cm^2^ for men and 19.9 cm^2^ for women.

### 2.5 Statistical analyses

All statistical analyses were conducted using EZR ver. 1.54,^19^ which is for R, and all *p*‐values were two‐sided. We used the two‐sided α level of 0.05. Our primary analyses were assessment of the survival association of postoperative CXI and dynamic change in CXI with disease‐free and overall survival. All other tests, including assessment of risk estimates, represented secondary analyses.

Data are expressed as median and interquartile range or number and ratio. Univariate analysis was performed using the Mann–Whitney *U*‐test, chi‐square test, or Fisher's exact test, as appropriate. Preoperative CXI cut‐off level for each sex was determined by maximizing the Youden index (the sum of sensitivity and specificity) for predicting 5‐year survival on each ROC curve among the patients who were followed up for over 5 years or who died within 5 years of follow‐up.

We investigated the relationship between clinicopathologic variables and disease‐free survival or overall survival after hepatic resection for HCC. Univariate and multivariate Cox proportional hazards regression models were used to estimate the hazard ratio for disease‐free and overall survival. The multivariable Cox regression model in Tables 2 and 3 initially included age (≥65 vs. <65 years), sex (female vs. male), hepatitis B surface antigen (HBsAg) status (positive vs. negative), hepatitis C virus antibody (HCV‐Ab) status (positive vs. negative), preoperative ICG_R15_ (≥15 vs. <15%), Child–Pugh grade (B vs. A), preoperative serum AFP level (≥20 vs. <20 ng/mL), preoperative PIVKA‐II level (≥200 vs. <200 mAU/mL), tumor differentiation (poor vs. well or moderate), tumor size (>5 vs. ≤5 cm), number of tumors (multiple vs. solitary), microvascular invasion (yes vs. no), type of resection (anatomical vs. partial), operation approach (open vs. laparoscopic), duration of operation (≥360 vs. <360 min), intraoperative blood loss (≥1000 vs. <1000 g), intraoperative blood transfusion (yes vs. no), postoperative complication (yes vs. no), sarcopenia (yes vs. no), preoperative CXI (low vs. high), and postoperative CXI (low vs. high). A backward elimination was conducted with a threshold *p* of 0.05 to select variables for the final models.

The Kaplan–Meier method was used to estimate cumulative survival probabilities, and the differences between groups were compared using the log‐rank test.

In the sensitivity analysis of clinicopathologic variables for survival after hepatic resection for HCC, the cut‐off value of postoperative CXI for each sex was determined based on the ROC analysis of survival status at 5‐year follow‐up in the same way as that of preoperative CXI. As an exploratory analysis, to evaluate the changes in the numerical value of CXI from before to after surgery, the ratio of postoperative CXI to preoperative CXI was calculated as an indicator of the changes in the numerical value of CXI from before to after surgery. We then investigated the prognostic value of this ratio using univariate and multivariate analyses.

## 3 RESULTS

### 3.1 Patient characteristics and univariate analysis of clinicopathological variables in relation to postoperative CXI

**FIGURE 1:**
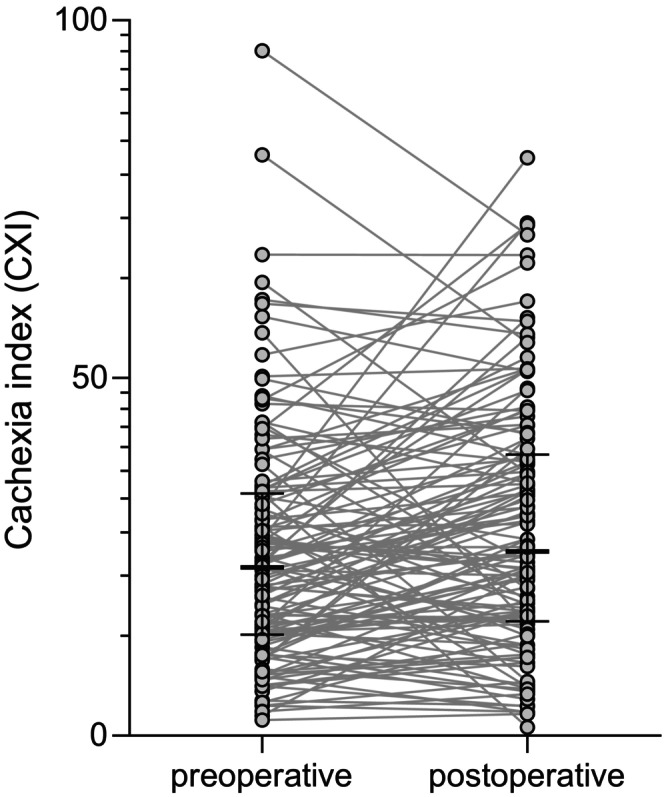
One‐dimensional scatter plots representing changes from preoperative to postoperative CXI (median with interquartile range). The median (interquartile range) values of preoperative and postoperative CXI were 23.5 (14.2–33.6) and 25.8 (16.0–39.2), respectively.

Clinicopathological characteristics of the enrolled patients are presented in Table 1 as median and interquartile range (IQR), or as number and ratio. The median (IQR) follow‐up period of 131 patients after hepatectomy was 5.2 (3.0–7.1) years. During this follow‐up, 83 of 131 patients experienced tumor recurrence (63.4%), and the median (IQR) time to recurrence following hepatic resection was 1.6 (0.7–3.0) years. At the time of postoperative CXI calculation, 10 of 131 patients had developed a recurrence. In the current study, the 5‐year disease‐free survival and overall survival rates after hepatic resection for HCC were 55.7% and 76.3%, respectively. The median (IQR) values of preoperative and postoperative CXI (Figure 1) were 23.5 (14.2–33.6) and 25.8 (16.0–39.2), respectively. Eighty of 131 patients (61%) increased their postoperative CXI compared to their preoperative CXI, while 51 patients (39%) decreased their postoperative CXI. Based on the ROC analysis of the survival status at the 5‐year follow‐up, the optimal cut‐off values of preoperative CXI for each sex (Figure S1) were set as 11.5 for men and 7.8 for women with areas under the curve of 0.599 (95% confidence interval (CI): 0.455–0.742) and 0.800 (95% CI: 0.468–1.00), respectively. Table 1 also shows the univariate analysis of clinicopathological variables in relation to postoperative CXI, of which the cut‐off values for each sex were the same as those of preoperative CXI. In the univariate analysis, low postoperative CXI was associated with older age (*p* = 0.045), greater tumor size (*p* < 0.01), longer operation time (*p* = 0.047), greater intraoperative bleeding (*p* < 0.01), intraoperative blood transfusion (*p* < 0.01), and low preoperative CXI (*p* < 0.01).

**TABLE 1:** Clinicopathological characteristics of enrolled patients and univariate analysis of clinicopathological variables in relation to postoperative cachexia index (CXI).

Variables	Total (*n* = 131)	Postoperative CXI		*p*‐value^a^
		High (*n* = 111)	Low (*n* = 20)	
Age, years	66 (61–74)	66 (61–74)	72 (65–78)	0.045
Sex				1.00
Female	19 (14.5%)	16 (14%)	3 (15%)	
Male	112 (85.5%)	95 (86%)	17 (85%)	
HBsAg, positive	28 (21%)	24 (22%)	4 (20%)	1.00
HCV‐Ab, positive	30 (23%)	22 (20%)	8 (40%)	0.08
Preoperative ICG_R15_, %	13 (9–21)	13 (9–21)	14 (9–20)	1.00
Child–Pugh grade, B	10 (8%)	8 (7%)	2 (10%)	0.65
Preoperative serum AFP level, ng/mL	10 (5–59)	9 (5–44)	33 (8–671)	0.06
Preoperative serum PIVKA‐II level, mAU/mL	69 (24–1610)	66 (24–1270)	767 (26–8523)	0.18
Tumor differentiation, poor	19 (15%)	16 (15%)	3 (15%)	1.00
Tumor size, cm	3.5 (2.5–6.8)	3.5 (2.2–5.5)	8.3 (3.3–10.5)	<0.01
Tumor number, multiple	32 (24%)	27 (24%)	5 (25%)	1.00
Microvascular invasion, yes	21 (16%)	18 (16%)	3 (15%)	1.00
Type of resection, anatomical	90 (69%)	75 (68%)	15 (75%)	0.61
Operation approach, open	115 (88%)	96 (86%)	19 (95%)	0.46
Duration of operation, min	409 (320–512)	388 (314–483)	460 (352–580)	0.047
Intraoperative blood loss, g	500 (250–1043)	460 (211–985)	893 (511–1633)	<0.01
Intraoperative BTF, yes	27 (21%)	18 (16%)	9 (45%)	<0.01
Postoperative complication, yes	46 (35%)	37 (33%)	9 (45%)	0.32
Sarcopenia, yes	64 (49%)	51 (46%)	13 (65%)	0.15
Preoperative CXI, low	22 (17%)	11 (10%)	11 (55%)	<0.01

Abbreviations: AFP, alpha‐fetoprotein; BTF, blood transfusion; CXI, cachexia index; HBsAg, hepatitis B surface antigen; HCV‐Ab, hepatitis C virus antibody; ICG_R15_, retention rate of indocyanine green at 15 min; PIVKA‐II, protein induced by vitamin K absence or antagonist‐II.

^a^ The chi‐square test was performed to compare categorical variables, while Mann–Whitney *U*‐test was performed to compare continuous variables.

### 3.2 Univariate and multivariate analyses of clinicopathological variables in relation to disease‐free survival after hepatic resection for HCC

**FIGURE 2:**
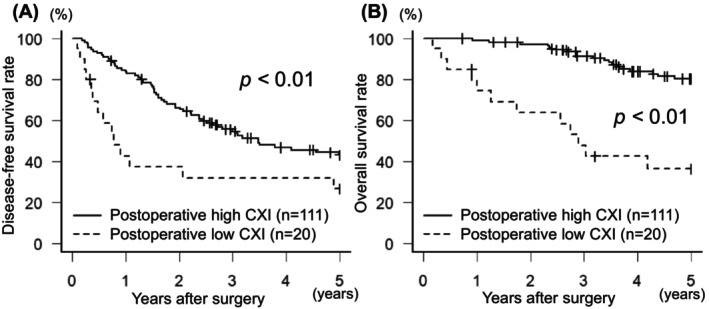
Kaplan–Meier curves of the disease‐free survival (A) and overall survival (B) after hepatic resection for hepatocellular carcinoma according to the status of postoperative cachexia index. Postoperative low cachexia index was significantly associated with worse disease‐free survival (*p* < 0.01) and overall survival (*p* < 0.01).

Table 2 shows the association between clinicopathological variables and disease‐free survival after hepatic resection for HCC. In the univariate analysis, disease‐free survival was significantly associated with HBsAg positivity (*p* < 0.01), preoperative serum PIVKA‐II level ≥ 200 mAU/mL (*p* < 0.01), poor tumor differentiation (*p* = 0.01), tumor size >5 cm (*p* < 0.01), multiple tumors (*p* < 0.01), microvascular invasion (*p* < 0.01), intraoperative blood transfusion (*p* = 0.03), postoperative complication (*p* < 0.01), low preoperative CXI (*p* < 0.01), and low postoperative CXI (*p* = 0.03, Figure 2A). In the multivariate analysis, HBsAg‐positivity (*p* = 0.02), preoperative serum PIVKA‐II level ≥ 200 mAU/mL (*p* < 0.01), poor tumor differentiation (*p* = 0.02), multiple tumors (*p* < 0.01), microvascular invasion (*p* < 0.01), type of resection (*p* < 0.01), postoperative complication (*p* < 0.01), and low preoperative CXI (*p* < 0.01) were independent and significant predictors of disease‐free survival.

**TABLE 2:** Univariate and multivariate analyses of prognostic factors for disease‐free survival in patients with hepatocellular carcinoma after hepatic resection.

Variables	Univariate analysis		Multivariate analysis	
	HR (95% CI)	*p*‐value	HR (95% CI)	*p*‐value^a^
Age, ≥ 65 years	1.19 (0.75–1.87)	0.46		NS
Sex, female	0.70 (0.35–1.40)	0.31		NS
HBsAg, positive	0.43 (0.23–0.80)	<0.01	0.47 (0.24–0.90)	0.02
HCV‐Ab, positive	1.06 (0.83–1.37)	0.64		NS
Preoperative ICG_R15_, ≥ 15%	1.25 (0.81–1.92)	0.32		NS
Child–Pugh grade, B	0.84 (0.34–2.09)	0.71		NS
Preoperative serum AFP level, ≥ 20 ng/mL	1.39 (0.90–2.17)	0.14		NS
Preoperative serum PIVKA‐II level, ≥ 200 mAU/mL	1.91 (1.23–2.96)	<0.01	2.52 (1.50–4.22)	<0.01
Tumor differentiation, poor	2.06 (1.18–3.60)	0.01	2.00 (1.11–3.60)	0.02
Tumor size, >5 cm	1.81 (1.16–2.83)	<0.01		NS
Tumor number, multiple	2.17 (1.37–3.44)	<0.01	3.17 (1.88–5.33)	<0.01
Microvascular invasion, yes	2.49 (1.47–4.24)	<0.01	2.77 (1.53–5.02)	<0.01
Type of resection, anatomical	0.80 (0.51–1.29)	0.33	0.44 (0.25–0.78)	<0.01
Operation approach, open	1.29 (0.62–2.69)	0.49		NS
Duration of operation, ≥ 360 min	1.03 (0.66–1.61)	0.89		NS
Intraoperative blood loss, ≥ 1000 g	1.49 (0.94–2.37)	0.09		NS
Intraoperative BTF, yes	1.76 (1.07–2.89)	0.03		NS
Postoperative complication, yes	1.88 (1.21–2.90)	<0.01	2.08 (1.27–3.39)	<0.01
Sarcopenia, yes	1.47 (0.96–2.27)	0.08		NS
Preoperative CXI, low	2.28 (1.32–3.95)	<0.01	3.99 (2.03–7.82)	<0.01
Postoperative CXI, low	1.84 (1.05–3.24)	0.03		NS

Abbreviations: AFP, alpha‐fetoprotein; BTF, blood transfusion; CI, confidence interval; CXI, cachexia index; HBsAg, hepatitis B surface antigen; HCV‐Ab, hepatitis C virus antibody; HR, hazard ratio; ICG_R15_, retention rate of indocyanine green at 15 min; PIVKA‐II, protein induced by vitamin K absence or antagonist‐II; NS, not significant.

^a^ The multivariable Cox regression model initially included age (≥ 65 vs. < 65 years), sex (female vs. male), HBsAg status (positive vs. negative), HCV‐Ab status (positive vs. negative), preoperative ICG_R15_ (≥ 15 vs. < 15%), Child–Pugh grade (B vs. A), preoperative serum AFP level (≥20 vs. <20 ng/mL), preoperative serum PIVKA‐II level (≥200 vs. <200 mAU/mL), tumor differentiation (poor vs. well or moderate), tumor size (>5 vs. ≤5 cm), number of tumors (multiple vs. solitary), microvascular invasion (yes vs. no), type of resection (anatomical vs. partial), operation approach (open vs. laparoscopic), duration of operation (≥360 vs. <360 min), intraoperative blood loss (≥1000 vs. <1000 g), intraoperative BTF (yes vs. no), postoperative complication (yes vs. no), sarcopenia (yes vs. no), preoperative CXI (low vs. high), and postoperative CXI (low vs. high). A backward elimination was conducted with a threshold *p* of 0.05 to select variables for the final models.

### 3.3 Univariate and multivariate analyses of clinicopathological variables in relation to overall survival after hepatic resection for HCC

Table 3 shows the association between clinicopathological variables and overall survival after hepatic resection for HCC. In the univariate analysis, overall survival was significantly associated with HBsAg‐positivity (*p* = 0.047), preoperative ICG_R15_ ≥ 15% (*p* = 0.04), tumor size >5 cm (*p* = 0.04), microvascular invasion (*p* = 0.04), intraoperative blood loss ≥1000 g (*p* = 0.048), intraoperative blood transfusion (*p* < 0.01), postoperative complication (*p* < 0.01), sarcopenia (*p* = 0.03), low preoperative CXI (*p* < 0.01), and low postoperative CXI (*p* < 0.01, Figure 2B). In the multivariate analysis, preoperative ICG_R15_ ≥ 15% (*p* = 0.01), poor tumor differentiation (*p* < 0.01), multiple tumors (*p* = 0.01), postoperative complication (*p* < 0.01), low preoperative CXI (*p* < 0.01), and low postoperative CXI (*p* < 0.01) were independent and significant predictors of overall survival.

**TABLE 3:** Univariate and multivariate analyses of prognostic factors for overall survival in patients with hepatocellular carcinoma after hepatic resection.

Variables	Univariate analysis		Multivariate analysis	
	HR (95% CI)	*p*‐value	HR (95% CI)	*p*‐value^a^
Age, ≥ 65 years	0.94 (0.50–1.78)	0.86		NS
Sex, female	0.69 (0.25–1.94)	0.48		NS
HBsAg, positive	0.39 (0.15–0.99)	0.047		NS
HCV‐Ab, positive	1.31 (0.94–1.82)	0.12		NS
Preoperative ICG_R15_, ≥ 15%	1.96 (1.04–3.69)	0.04	2.46 (1.24–4.91)	0.01
Child‐Pugh grade, B	1.67 (0.59–4.73)	0.33		NS
Preoperative serum AFP level, ≥ 20 ng/mL	1.69 (0.90–3.17)	0.10		NS
Preoperative serum PIVKA‐II level, ≥ 200 mAU/mL	1.50 (0.80–2.82)	0.20		NS
Tumor differentiation, poor	2.01 (0.95–4.24)	0.07	3.85 (1.70–8.76)	<0.01
Tumor size, >5 cm	1.92 (1.02–3.60)	0.04		NS
Tumor number, multiple	1.79 (0.93–3.43)	0.08	2.43 (1.20–4.91)	0.01
Microvascular invasion, yes	2.08 (1.02–4.27)	0.04		NS
Type of resection, anatomical	0.89 (0.46–1.70)	0.71		NS
Operation approach, open	2.05 (0.49–8.52)	0.32		NS
Duration of operation, ≥ 360 min	1.21 (0.63–2.31)	0.57		NS
Intraoperative blood loss, ≥ 1000 g	1.89 (1.00–3.56)	0.048		NS
Intraoperative BTF, yes	2.74 (1.44–5.21)	<0.01		NS
Postoperative complication, yes	2.51 (1.34–4.69)	<0.01	2.89 (1.47–5.68)	<0.01
Sarcopenia, yes	2.05 (1.08–3.89)	0.03		NS
Preoperative CXI, low	3.29 (1.63–6.64)	<0.01	3.46 (1.48–8.09)	<0.01
Postoperative CXI, low	3.56 (1.80–7.03)	<0.01	3.88 (1.74–8.66)	<0.01

Abbreviations: AFP, alpha‐fetoprotein; BTF, blood transfusion; CI, confidence interval; CXI, cachexia index; HBsAg, hepatitis B surface antigen; HCV‐Ab, hepatitis C virus antibody; HR, hazard ratio; ICG_R15_, retention rate of indocyanine green at 15 min; PIVKA‐II, protein induced by vitamin K absence or antagonist‐II; NS, not significant.

^a^ The multivariable Cox regression model initially included age (≥65 vs. <65 years), sex (female vs. male), HBsAg status (positive vs. negative), HCV‐Ab status (positive vs. negative), preoperative ICG_R15_ (≥15 vs. <15%), Child–Pugh grade (B vs. A), preoperative serum AFP level (≥20 vs. <20 ng/mL), preoperative serum PIVKA‐II level (≥200 vs. <200 mAU/mL), tumor differentiation (poor vs. well or moderate), tumor size (>5 vs. ≤5 cm), number of tumors (multiple vs. solitary), microvascular invasion (yes vs. no), type of resection (anatomical vs. partial), operation approach (open vs. laparoscopic), duration of operation (≥360 vs. <360 min), intraoperative blood loss (≥1000 vs. <1000 g), intraoperative BTF (yes vs. no), postoperative complication (yes vs. no), sarcopenia (yes vs. no), preoperative CXI (low vs. high), and postoperative CXI (low vs. high). A backward elimination was conducted with a threshold *p* of 0.05 to select variables for the final models.

### 3.4 Survival according to dynamic changes in CXI from before to after hepatic resection for HCC, and clinicopathological variables affecting the dynamic changes in CXI

**FIGURE 3:**
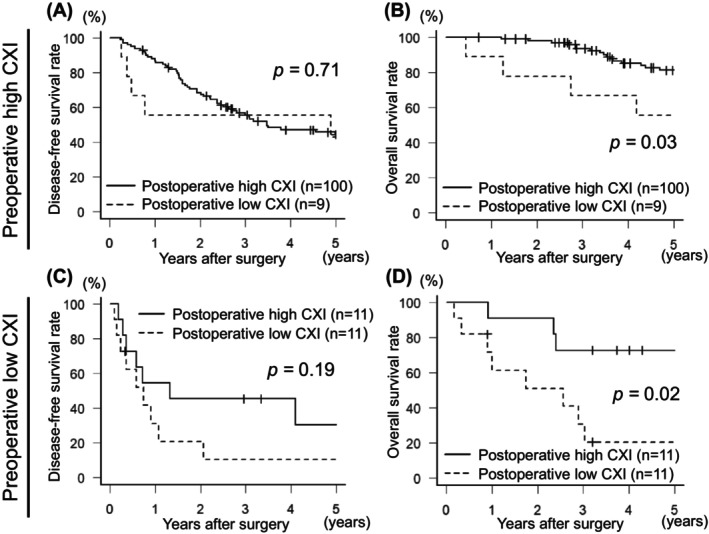
Kaplan–Meier curves of the disease‐free and overall survival after hepatic resection for hepatocellular carcinoma according to the status of postoperative cachexia index in preoperative high (A and B) or low (C and D) cachexia index group. Postoperative low cachexia index was significantly associated with worse overall survival both in preoperative high cachexia index group (*p* = 0.03) and in preoperative low cachexia index group (*p* = 0.02).

We assessed the association of dynamic changes in CXI from before to after hepatic resection for HCC with disease‐free survival and overall survival, and investigated clinicopathological variables affecting the dynamic changes in CXI. Among patients with preoperative high CXI, although there was no statistical difference in disease‐free survival (*p* = 0.71, Figure 3A), patients with a change from high preoperative CXI to low postoperative CXI had significantly worse overall survival than those with a maintained high CXI from before to after surgery (*p* = 0.03, Figure 3B). A change from high preoperative CXI to low postoperative CXI was significantly associated with longer operation time (*p* = 0.04), greater intraoperative bleeding (*p* < 0.01), and intraoperative blood transfusion (*p* < 0.01) (Table 4). Among patients with low preoperative CXI, patients with a change from low preoperative CXI to high postoperative CXI had a tendency toward better disease‐free survival (*p* = 0.19, Figure 3C) and had significantly better overall survival compared to those with a maintained low CXI from before to after surgery (*p* = 0.03, Figure 3D). Older age was significantly associated with a change from low preoperative CXI to high postoperative CXI (*p* < 0.01) (Table 4). Among the patients with low postoperative CXI, patients with high preoperative CXI showed better disease‐free and overall survival compared with those in patients with low preoperative CXI. Also, among the patients with high postoperative CXI, patients with high preoperative CXI showed better disease‐free and overall survival compared with that in patients with low preoperative CXI (Figure S2). Regardless of preoperative CXI status, patients with high postoperative CXI tended to receive repeated hepatic resection as a treatment for recurrence of HCC, while patients who maintained low CXI from before to after surgery tended to receive best supportive care (Table 4). Due to the limited number of cases, there were no significant differences between the four subgroups classified by the status of preoperative and postoperative CXI. However, differences in overall survival were observed according to treatment after recurrence in both high postoperative CXI group (*p* = 0.01) and low postoperative CXI group (*p* = 0.02) (Figure S3). In both the high and low postoperative CXI groups, patients who received repeated hepatic resection or radiofrequency ablation (RFA) had significantly better overall survival compared to those who received chemotherapy, TACE, or best supportive care (*p* < 0.01, Figure S3).

**TABLE 4:** Patient characteristics according to the status of postoperative CXI in high preoperative CXI group and low preoperative CXI group.

Variables	Post CXI in pre CXI‐high‐group		*p*‐value^a^	Post CXI in pre CXI‐low‐group		*p*‐value^a^
	High	Low		High	Low	
	(*n* = 100)	(*n* = 9)		(*n* = 11)	(*n* = 11)	
Age, years	66 (61–74)	66 (60–76)	0.92	65 (49–69)	74 (71–81)	<0.01
Gender			0.61			0.59
Female	13 (13%)	2 (22%)		3 (27%)	1 (9%)	
Male	87 (87%)	7 (78%)		8 (73%)	10 (91%)	
HBsAg, positive	21 (21%)	3 (33%)	0.41	3 (27%)	1 (9%)	0.59
HCV‐Ab, positive	21 (21%)	4 (44%)	0.21	1 (9%)	4 (36%)	0.31
Preoperative ICG_R15_, %	14 (10–21)	16 (9–26)	0.69	8.0 (5.5–10.5)	10 (9.0–18.5)	0.08
Child–Pugh grade, B	6 (6%)	0 (0%)	1.00	2 (18%)	2 (18%)	1.00
Preoperative serum AFP level, ng/mL	8 (5–41)	19 (9–234)	0.15	12 (4–1727)	44 (8–727)	0.69
Preoperative serum PIVKA‐II level, mAU/mL	68 (25–1428)	559 (26–1120)	0.56	51 (20–630)	1390 (26–14 570)	0.26
Tumor differentiation, poor	15 (15%)	0 (0%)	0.35	1 (9%)	3 (27%)	0.59
Tumor size, cm	3.3 (2.2–5.0)	3.3 (3.0–9.0)	0.32	7.6 (4.5–14.0)	9.5 (7.4–13.0)	0.51
Tumor number, multiple	23 (23%)	4 (44%)	0.22	4 (36%)	1 (9%)	0.31
Microvascular invasion, yes	13 (13%)	1 (11%)	1.00	5 (50%)	2 (18%)	0.18
Type of resection, anatomical	64 (64%)	7 (78%)	0.49	11 (100%)	8 (73%)	0.21
Operation approach, open	85 (85%)	9 (100%)	0.36	11 (100%)	10 (91%)	1.00
Duration of operation, min	399 (318–487)	517 (452–566)	0.04	341 (269–406)	419 (337–584)	0.19
Intraoperative blood loss, g	443 (202–933)	975 (750–2440)	<0.01	650 (238–1355)	810 (493–1495)	0.65
Intraoperative BTF, yes	15 (15%)	5 (56%)	<0.01	3 (27%)	4 (36%)	1.00
Postoperative complication, yes	32 (32%)	2 (22%)	0.72	5 (46%)	7 (64%)	0.67
Sarcopenia, yes	44 (44%)	3 (33%)	0.73	7 (64%)	10 (91%)	0.31
Treatment for recurrence			0.39			0.32
Repeated hepatic resection	27 (44%)	1 (17%)		3 (43%)	1 (11%)	
Radiofrequency ablation	10 (16%)	2 (33%)		0 (0%)	1 (11%)	
Chemotherapy	4 (7%)	1 (17%)		1 (14%)	1 (11%)	
TACE	15 (25%)	2 (33%)		3 (43%)	3 (33%)	
Best Supportive care	5 (8%)	0 (0%)		0 (0%)	3 (33%)	

Abbreviations: AFP, alpha‐fetoprotein; BTF, blood transfusion; CXI, cachexia index; HBsAg, hepatitis B surface antigen; HCV‐Ab, hepatitis C virus antibody; ICG_R15_, retention rate of indocyanine green at 15 min; PIVKA‐II, protein induced by vitamin K absence or antagonist‐II; Post CXI, postoperative cachexia index; Pre CXI, preoperative cachexia index; TACE, transcatheter arterial chemoembolization.

^a^ The chi‐square test was performed to compare categorical variables, while Mann–Whitney *U*‐test was performed to compare continuous variables.

### 3.5 Preoperative CXI, postoperative CXI, and ratio of perioperative change in CXI and survival after hepatic resection for HCC

As a sensitivity analysis, we investigated the prognostic value of postoperative CXI using the different cut‐off values of preoperative CXI. Similar to the cut‐off values of preoperative CXI, based on the ROC analysis of survival status at 5‐year follow‐up, the optimal cut‐off values of postoperative CXI for each sex (Figure S4) were determined to be 11.4 for men and 19.0 for women, with areas under the curve of 0.603 (95% CI: 0.458–0.748) and 0.600 (95% CI: 0.154–1.00), respectively. When postoperative CXI was classified into the two groups using these cut‐off values, postoperative low CXI had a trend toward worse disease‐free survival (*p* = 0.09), and was significantly associated with worse overall survival (*p* < 0.01) (Figure S5).

As an exploratory analysis, the ratio of postoperative CXI to preoperative CXI was calculated and we investigated the prognostic value of this ratio. The median (IQR) value of the ratio of perioperative change in CXI, the ratio of postoperative CXI to preoperative CXI, was 1.19 (0.85–1.60). The cut‐off value of the ratio of perioperative change in CXI was determined to be 0.50 based on the median absolute distance from 1.00 of the ratios of postoperative CXI to preoperative CXI. Low ratio of perioperative change in CXI was significantly associated with worse disease‐free survival (*p* < 0.01) and overall survival (*p* = 0.03) (Figure S6). In the multivariate analysis, low ratio of perioperative change in CXI was significantly associated with worse disease‐free (*p* < 0.01, Table S1) and overall survival (*p* < 0.01, Table S2).

## 4 DISCUSSION

In the present study, we found that postoperative CXI and dynamic changes in CXI from before to after hepatic resection were significantly associated with overall survival in patients with HCC. Interestingly, intraoperative blood loss in the preoperative high CXI group and age in the preoperative low CXI group were associated with low postoperative CXI. Moreover, the ratio of perioperative change in CXI was associated with both disease‐free and overall survival. Our findings indicated the potential of dynamic change in CXI from before to after surgery to enable the fine risk stratification of patients, leading to the development of a personalized treatment strategy for HCC.

Underlying the pathogenesis of cachexia are many inflammatory cytokines such as tumor necrosis factor‐alpha (TNF‐α), interleukin‐1beta (IL‐1β), and interleukin‐6 (IL‐6), which cause malnutrition and loss of skeletal muscle mass.^20^, ^21^ Given these inflammatory cytokines are associated with increased NLR,^22^ CXI consisting of skeletal muscle index, NLR, and serum albumin level, may be associated with these inflammatory cytokines. Surgery itself also induces these inflammatory cytokines through the activation of macrophages and neutrophils.^23^ The majority of postoperative patients have inadequate protein and energy intake.^24^ Postoperative complications cause a prolonged systemic inflammatory response, decreased nutritional intake, and delayed ambulation,^3^, ^10^ and sustained inflammation causes skeletal muscle wasting^20^ and persistent anorexia.^21^ Therefore, surgical stress may worsen the degree of preoperative cachexia or contribute to developing postoperative cachexia. Since postoperative cachexia, represented by low postoperative CXI, was associated with poor overall survival, perioperative exacerbation of cachexia should be avoided. Perioperative risk stratification based on cachexia may allow for an improved treatment strategy for HCC.

In this study, patients with low CXI preoperatively but who improved their CXI postoperatively were younger than those who did not improve their low CXI until postoperatively. Surgical resection of tumors can potentially improve cancer cachexia because it removes the tumors producing inflammatory cytokines that cause the status of cachexia. Given aging leads to delayed wound healing, increased susceptibility to infection, loss of muscle quality, and chronic inflammation called inflammaging,^25^, ^26^ it may be difficult for older people to improve their cachexia even after tumor removal. Therefore, older people with preoperative cachexia may require more intensive preoperative nutritional and physical therapy to improve their cachexia. In patients with severe nutritional risk, nutritional support for 10–14 days prior to major surgery and the addition of oral nutritional supplements to the regular diet both during hospitalization and after discharge have been recommended.^27^ Long‐chain N‐3 fatty acids have reduced inflammatory cytokines such as IL‐6 or C‐reactive protein and resting energy expenditure in cancer patients.^27^ Both preoperative and postoperative exercise interventions have been shown to increase a patient's muscle mass.^27^, ^28^ CXI can potentially reflect the effect of these interventions on patient's condition and might be a useful biomarker that contributes to comprehensive evaluation. On the other hand, many of the patients with high CXI preoperatively but worsened CXI postoperatively had a greater degree of surgical stress, including longer operation time, greater intraoperative blood loss, and blood transfusion. Severe surgical stress results in the overproduction of inflammatory cytokines,^23^ which may become the trigger for cachexia. In fact, excessive intraoperative blood loss is undesirable because it leads to prolonged operation time and blood transfusion, and is associated with poor prognosis of HCC.^29^ For patients without preoperative cachexia, massive blood loss should be avoided as much as possible to prevent the formation of postoperative cachexia. A previous study has shown that laparoscopic hepatic resection was superior to open hepatic resection regarding intraoperative blood loss and blood transfusion rate.^30^ Minimally invasive surgery can potentially contribute to maintaining the CXI. Furthermore, for patients with low postoperative CXI, nutritional and physical intervention is required to improve their prognosis.

This study had several limitations. First, there was a potential risk of selection bias because this study was a retrospective review. Second, given the number of subjects in the subgroup analysis of preoperative CXI, the statistical power to investigate the effects of dynamic changes in CXI on survival was limited. More comprehensive large‐scale studies are expected to validate our findings. Third, postoperative CXI in this study was evaluated at approximately 3 months after liver resection for HCC as the first surveillance is usually performed at 3 months. However, skeletal muscle can change in the early operative period and the serial change of the skeletal muscle in the perioperative period is unknown. Therefore, further studies are needed to evaluate CXI at serial time points including the perioperative period and later than 3 months after surgery. Fourth, the effect of preoperative interventions to improve patient conditions could not be evaluated in this retrospective study. As several studies indicated that nutritional and physical interventions can improve cancer cachexia,^27^, ^28^ further prospective studies are necessary to confirm whether the improvement of CXI by interventions can contribute to a better prognosis.

In conclusion, not only postoperative CXI but also dynamic changes in CXI from pre‐ to post‐hepatectomy can be a prognostic indicator in patients with HCC after hepatic resection. Furthermore, surgical stress in patients with high preoperative CXI and age in patients with low preoperative CXI may be associated with lower postoperative CXI, leading to worse overall survival.

## AUTHOR CONTRIBUTIONS

MA and KH developed the main concept and designed the study. YY, KO, KF, MT, YS, SO, and MM were responsible for acquisition of clinicopathological data. MA and KH performed data analysis and interpretation. MA, KH, and TI drafted the manuscript. YY, KO, KF, MT, YS, SO, and MM contributed to editing and critical revision for important intellectual contents.

## FUNDING INFORMATION

This work was supported by JSPS KAKENHI grant numbers JP21K08718 (to T.I.) and JP21K08805 (to K.H.) and by research grants from the Yakult Bio‐Science Foundation (to K.H.) and Takeda Science Foundation (to K.H.). The funders had no role in study design, data collection and analysis, decision to publish, or preparation of the manuscript.

## CONFLICT OF INTEREST STATEMENT

The authors declare no conflict of interests for this article.

## ETHICS STATEMENTS

Ethics approval: This study protocol was approved by the Ethics Committee of the Jikei University School of Medicine (approval number: 27–177).

Informed consent: Individual consent was waived because of the anonymous nature of the data.

Registry and the registration no. of the study/trial: Not applicable.

Animal studies: Not applicable.

## Supporting information


Figure S1.
Figure S2.
Figure S3.
Figure S4.
Figure S5.
Figure S6.
Table S1.
Table S2.

## References

[1] Sung H , Ferlay J , Siegel RL , Laversanne M , Soerjomataram I , Jemal A , et al. Global cancer statistics 2020: GLOBOCAN estimates of incidence and mortality worldwide for 36 cancers in 185 countries. CA Cancer J Clin. 2021;71:209–249. 33538338 10.3322/caac.21660

[2] Gelli M , Sebagh M , Porcher R , Romanelli E , Vibert E , Sa Cunha A , et al. Liver resection for early hepatocellular carcinoma: preoperative predictors of non transplantable recurrence and implications for treatment allocation. Ann Surg. 2020;272:820–826. 32833755 10.1097/SLA.0000000000004259

[3] Haruki K , Taniai T , Yanagaki M , Furukawa K , Tsunematsu M , Onda S , et al. Sustained systemic inflammatory response predicts survival in patients with hepatocellular carcinoma after hepatic resection. Ann Surg Oncol. 2023;30:604–613. 36059035 10.1245/s10434-022-12464-6

[4] Fearon K , Strasser F , Anker SD , Bosaeus I , Bruera E , Fainsinger RL , et al. Definition and classification of cancer cachexia: an international consensus. Lancet Oncol. 2011;12:489–495. 21296615 10.1016/S1470-2045(10)70218-7

[5] Rich NE , Phen S , Desai N , Mittal S , Yopp AC , Yang JD , et al. Cachexia is prevalent in patients with hepatocellular carcinoma and associated with worse prognosis. Clin Gastroenterol Hepatol. 2022;20:e1157–e1169. 34555519 10.1016/j.cgh.2021.09.022 PMC8934317

[6] Go SI , Park MJ , Lee GW . Clinical significance of the cachexia index in patients with small cell lung cancer. BMC Cancer. 2021;21:563. 34001060 10.1186/s12885-021-08300-x PMC8130111

[7] Tanji Y , Furukawa K , Haruki K , Taniai T , Onda S , Tsunematsu M , et al. Significant impact of cachexia index on the outcomes after hepatic resection for colorectal liver metastases. Ann Gastroenterol Surg. 2022;6:804–812. 36338593 10.1002/ags3.12578 PMC9628226

[8] Karmali R , Alrifai T , Fughhi IAM , Ng R , Chukkapalli V , Shah P , et al. Impact of cachexia on outcomes in aggressive lymphomas. Ann Hematol. 2017;96:951–956. 28417157 10.1007/s00277-017-2958-1

[9] Akaoka M , Haruki K , Taniai T , Yanagaki M , Igarashi Y , Furukawa K , et al. Clinical significance of cachexia index in patients with hepatocellular carcinoma after hepatic resection. Surg Oncol. 2022;45:101881. 36371905 10.1016/j.suronc.2022.101881

[10] Choi MH , Yoon SB , Lee K , Song M , Lee IS , Lee MA , et al. Preoperative sarcopenia and post‐operative accelerated muscle loss negatively impact survival after resection of pancreatic cancer. J Cachexia Sarcopenia Muscle. 2018;9:326–334. 29399990 10.1002/jcsm.12274 PMC5879976

[11] Ocuin LM , Tsung A . Minimally invasive hepatic surgery. Surg Clin North Am. 2016;96:299–313. 27017866 10.1016/j.suc.2015.12.004

[12] Miyagawa S , Makuuchi M , Kawasaki S , Kakazu T . Criteria for safe hepatic resection. Am J Surg. 1995;169:589–594. 7771622 10.1016/s0002-9610(99)80227-x

[13] Wakabayashi G , Cherqui D , Geller DA , Abu Hilal M , Berardi G , Ciria R , et al. The Tokyo 2020 terminology of liver anatomy and resections: updates of the Brisbane 2000 system. J Hepatobiliary Pancreat Sci. 2022;29:6–15. 34866349 10.1002/jhbp.1091

[14] Haruki K , Furukawa K , Fujiwara Y , Taniai T , Hamura R , Shirai Y , et al. Effectiveness of anatomical resection for small hepatocellular carcinoma: a propensity score‐matched analysis of a multi‐institutional database. J Gastrointest Surg. 2021;25:2835–2841. 33772400 10.1007/s11605-021-04985-4

[15] Ueno S , Tanabe G , Nuruki K , Hamanoue M , Komorizono Y , Oketani M , et al. Prognostic performance of the new classification of primary liver cancer of Japan (4th edition) for patients with hepatocellular carcinoma: a validation analysis. Hepatol Res. 2002;24:395–403. 12479938 10.1016/s1386-6346(02)00144-4

[16] Dindo D , Demartines N , Clavien PA . Classification of surgical complications: a new proposal with evaluation in a cohort of 6336 patients and results of a survey. Ann Surg. 2004;240:205–213. 15273542 10.1097/01.sla.0000133083.54934.ae PMC1360123

[17] Wang Y , Peng C , Cheng Z , Wang X , Wu L , Li J , et al. The prognostic significance of preoperative neutrophil‐lymphocyte ratio in patients with hepatocellular carcinoma receiving hepatectomy: a systematic review and meta‐analysis. Int J Surg. 2018;55:73–80. 29787804 10.1016/j.ijsu.2018.05.022

[18] Masuda T , Shirabe K , Ikegami T , Harimoto N , Yoshizumi T , Soejima Y , et al. Sarcopenia is a prognostic factor in living donor liver transplantation. Liver Transpl. 2014;20:401–407. 24357065 10.1002/lt.23811

[19] Kanda Y . Investigation of the freely available easy‐to‐use software ‘EZR’ for medical statistics. Bone Marrow Transplant. 2013;48:452–458. 23208313 10.1038/bmt.2012.244 PMC3590441

[20] Argilés JM , Busquets S , Stemmler B , López‐Soriano FJ . Cancer cachexia: understanding the molecular basis. Nat Rev Cancer. 2014;14:754–762. 25291291 10.1038/nrc3829

[21] Suzuki H , Asakawa A , Amitani H , Nakamura N , Inui A . Cancer cachexia—pathophysiology and management. J Gastroenterol. 2013;48:574–594. 23512346 10.1007/s00535-013-0787-0 PMC3698426

[22] de Castro SI , Bianchi A , Deshpande NU , et al. Neutrophil‐mediated fibroblast‐tumor cell il‐6/stat‐3 signaling underlies the association between neutrophil‐to‐lymphocyte ratio dynamics and chemotherapy response in localized pancreatic cancer: a hybrid clinical‐preclinical study. Elife. 2022;11:e78921. 36107485 10.7554/eLife.78921 PMC9512403

[23] Shakhar G , Ben‐Eliyahu S . Potential prophylactic measures against postoperative immunosuppression: could they reduce recurrence rates in oncological patients? Ann Surg Oncol. 2003;10:972–992. 14527919 10.1245/aso.2003.02.007

[24] Constansia RDN , Hentzen JEKR , Hogenbirk RNM , van der Plas WY , Campmans‐Kuijpers MJE , Buis CI , et al. Actual postoperative protein and calorie intake in patients undergoing major open abdominal cancer surgery: a prospective, observational cohort study. Nutr Clin Pract. 2022;37:183–191. 33979002 10.1002/ncp.10678 PMC9292321

[25] den Braber I , Mugwagwa T , Vrisekoop N , Westera L , Mögling R , Bregje de Boer A , et al. Maintenance of peripheral naïve T cells is sustained by thymus output in mice but not humans. Immunity. 2012;36:288–297. 22365666 10.1016/j.immuni.2012.02.006

[26] Franceschi C , Garagnani P , Parini P , Giuliani C , Santoro A . Inflammaging: a new immune‐metabolic viewpoint for age‐related diseases. Nat Rev Endocrinol. 2018;14:576–590. 30046148 10.1038/s41574-018-0059-4

[27] Arends J , Bachmann P , Baracos V , Barthelemy N , Bertz H , Bozzetti F , et al. ESPEN guidelines on nutrition in cancer patients. Clin Nutr. 2017;36:11–48. 27637832 10.1016/j.clnu.2016.07.015

[28] Yamamoto K , Nagatsuma Y , Fukuda Y , Hirao M , Nishikawa K , Miyamoto A , et al. Effectiveness of a preoperative exercise and nutritional support program for elderly sarcopenic patients with gastric cancer. Gastric Cancer. 2017;20:913–918. 28032232 10.1007/s10120-016-0683-4

[29] Katz SC , Shia J , Liau KH , Gonen M , Ruo L , Jarnagin WR , et al. Operative blood loss independently predicts recurrence and survival after resection of hepatocellular carcinoma. Ann Surg. 2009;249:617–623. 19300227 10.1097/SLA.0b013e31819ed22f

[30] Xiangfei M , Yinzhe X , Yingwei P , Shichun L , Weidong D . Open versus laparoscopic hepatic resection for hepatocellular carcinoma: a systematic review and meta‐analysis. Surg Endosc. 2019;33:2396–2418. 31139980 10.1007/s00464-019-06781-3

