# Supplementary material for: The prognostic impact of perioperative dynamic changes in cachexia index in patients with hepatocellular carcinoma
Source: Ann Gastroenterol Surg. 2024 Apr 16;8(5):917–26. doi: 10.1002/ags3.12804 (PMC11368508; doi:10.1002/ags3.12804)
Supplement: Supplementary file 1 — Figure S1. Figure S2. Figure S3. Figure S4. Figure S5. Figure S6. Table S1. Table S2. [file AGS3-8-917-s001.docx]

**Supplementary Figure 1**

**
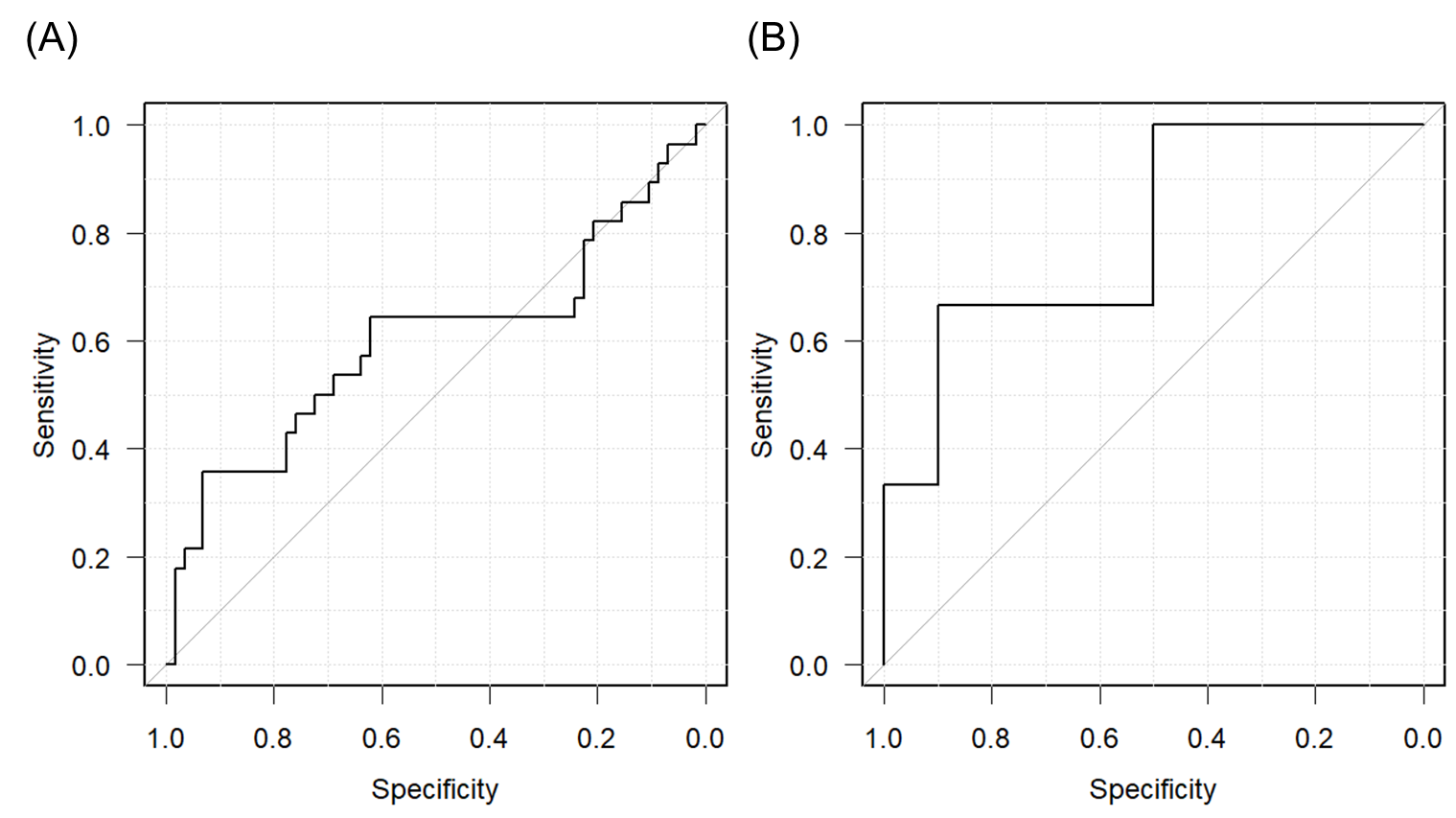
**

Receiver operating characteristic curve of the respective male’s preoperative cachexia index (CXI) (A) and female’s preoperative CXI (B) for survival status at 5-year follow-up. The optimal cut-off values for male’s preoperative CXI and female’s preoperative CXI were 11.5 and 7.8, with areas under the curve of 0.599 (95% confidence interval (CI): 0.455-0.742) and 0.800 (95% CI: 0.468-1.00), respectively.

**Supplementary Figure 2**


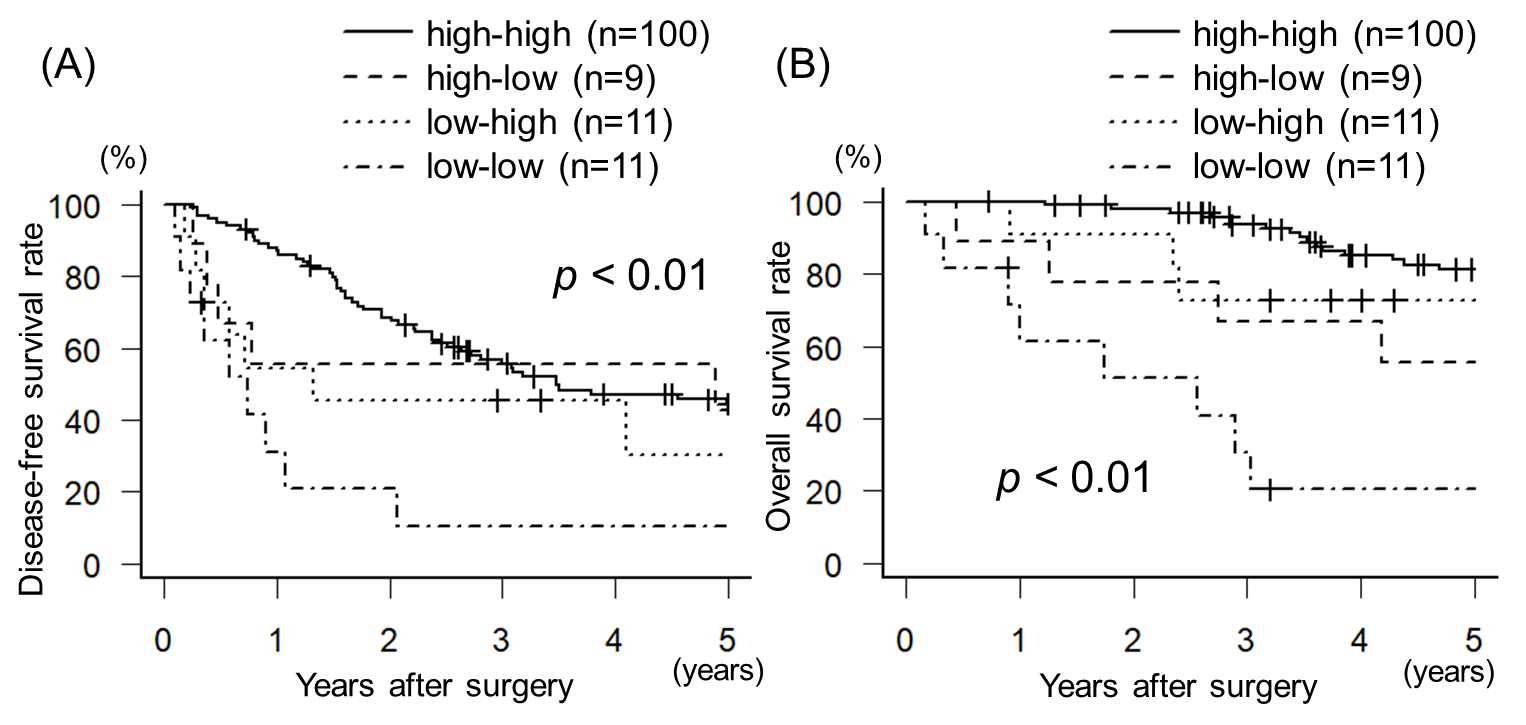


Kaplan–Meier curves of the disease-free survival (A) and overall survival (B) after hepatic resection for hepatocellular carcinoma according to perioperative changes in CXI classified by the status of preoperative cachexia index (CXI) and postoperative CXI (preoperative CXI’s status – postoperative CXI’s status).

Both disease-free survival and overall survival differed by perioperative changes in CXI (*p*<0.01).

**Supplementary Figure 3**


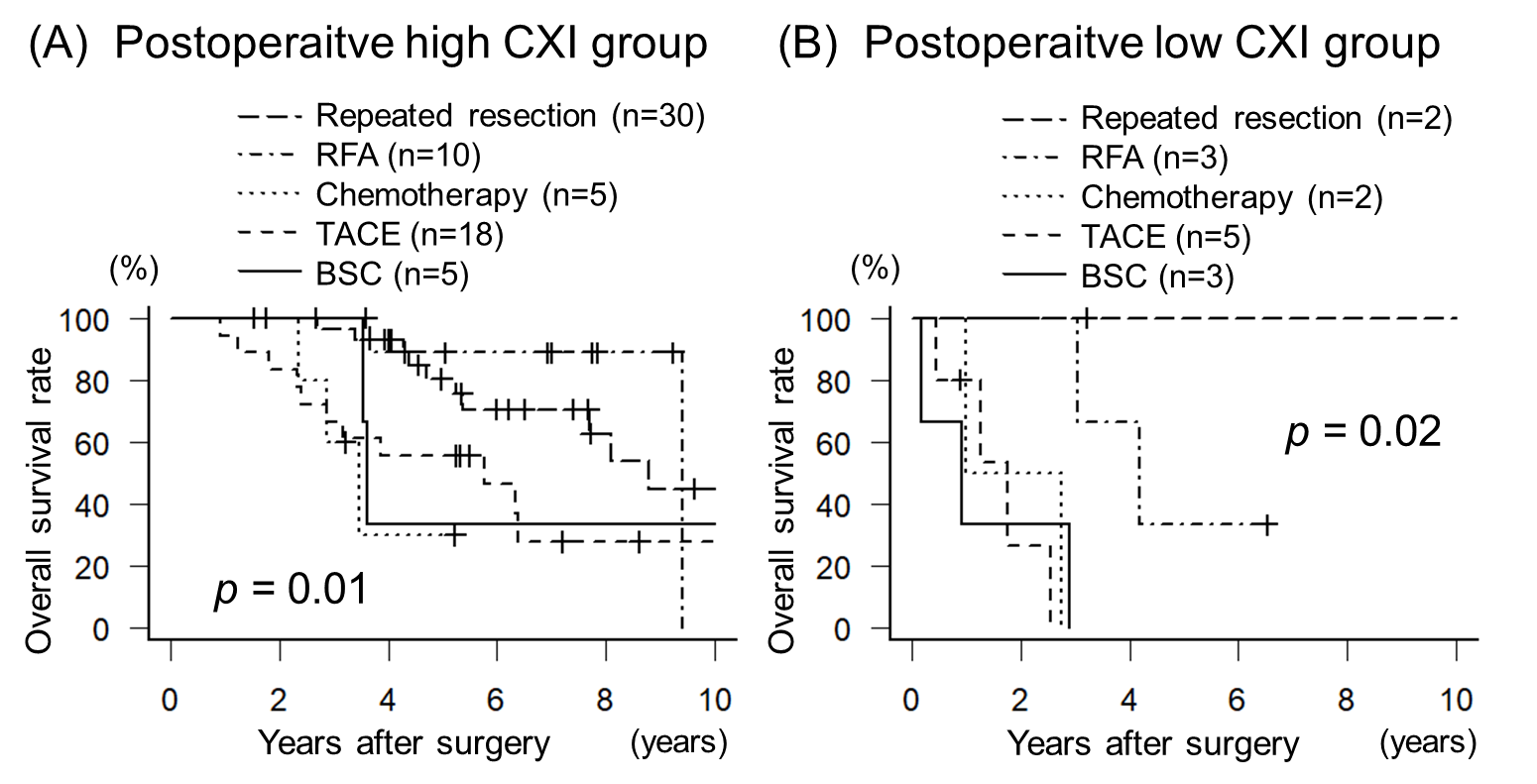


Kaplan-Meier curves of overall survival after hepatic resection for hepatocellular carcinoma according to treatment for recurrence after resection in postoperative high (A) or low (B) cachexia index (CXI) group.

In both the high and low postoperative CXI groups, patients who received repeated hepatic resection or radiofrequency ablation (RFA) had significantly better overall survival compared to those who received other treatment or best supportive care (BSC) (*p*<0.01).

**Supplementary Figure 4**

**
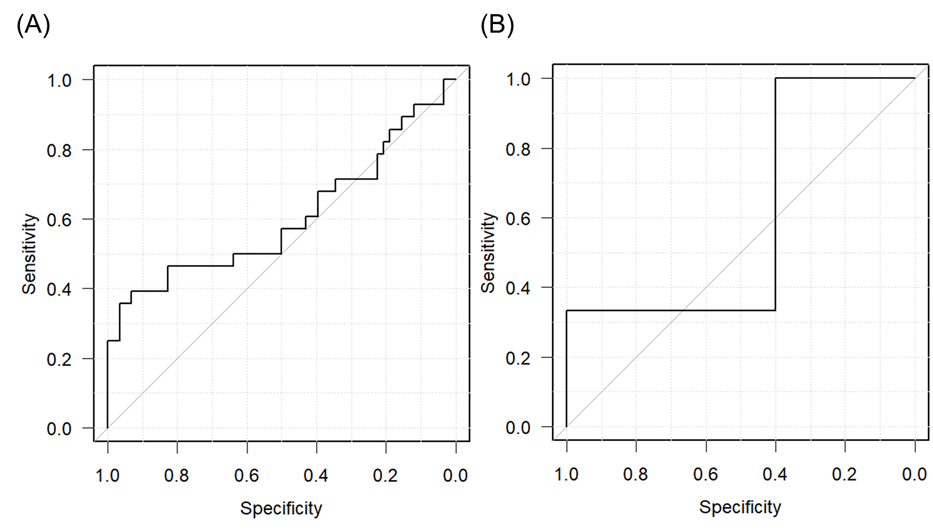
**

Receiver operating characteristic curve of the respective male’s postoperative cachexia index (CXI) (A) and female’s postoperative CXI (B) for survival status at 5-year follow-up. The optimal cut-off values for male’s postoperative CXI and female’s postoperative CXI were 11.4 and 19.0, with areas under the curve of 0.603 (95% confidence interval (CI): 0.458-0.748) and 0.600 (95% CI: 0.154-1.00), respectively.

**Supplementary Figure 5**


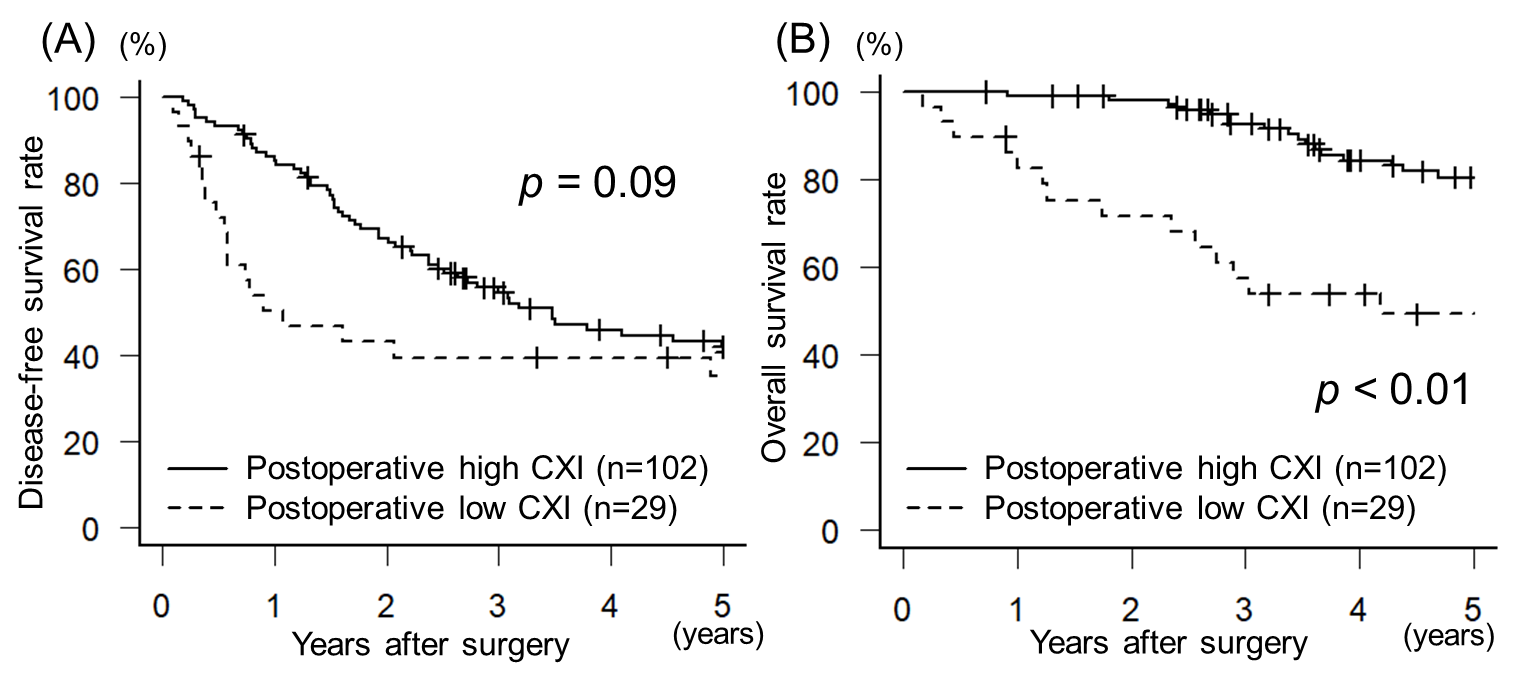


Kaplan-Meier curves of disease-free survival (A) and overall survival (B) after hepatic resection for hepatocellular carcinoma according to postoperative cachexia index (CXI) status. Postoperative low CXI had a trend toward worse disease-free survival (*p*=0.09), and was significantly associated with worse overall survival (*p*<0.01).

**Supplementary Figure 6**


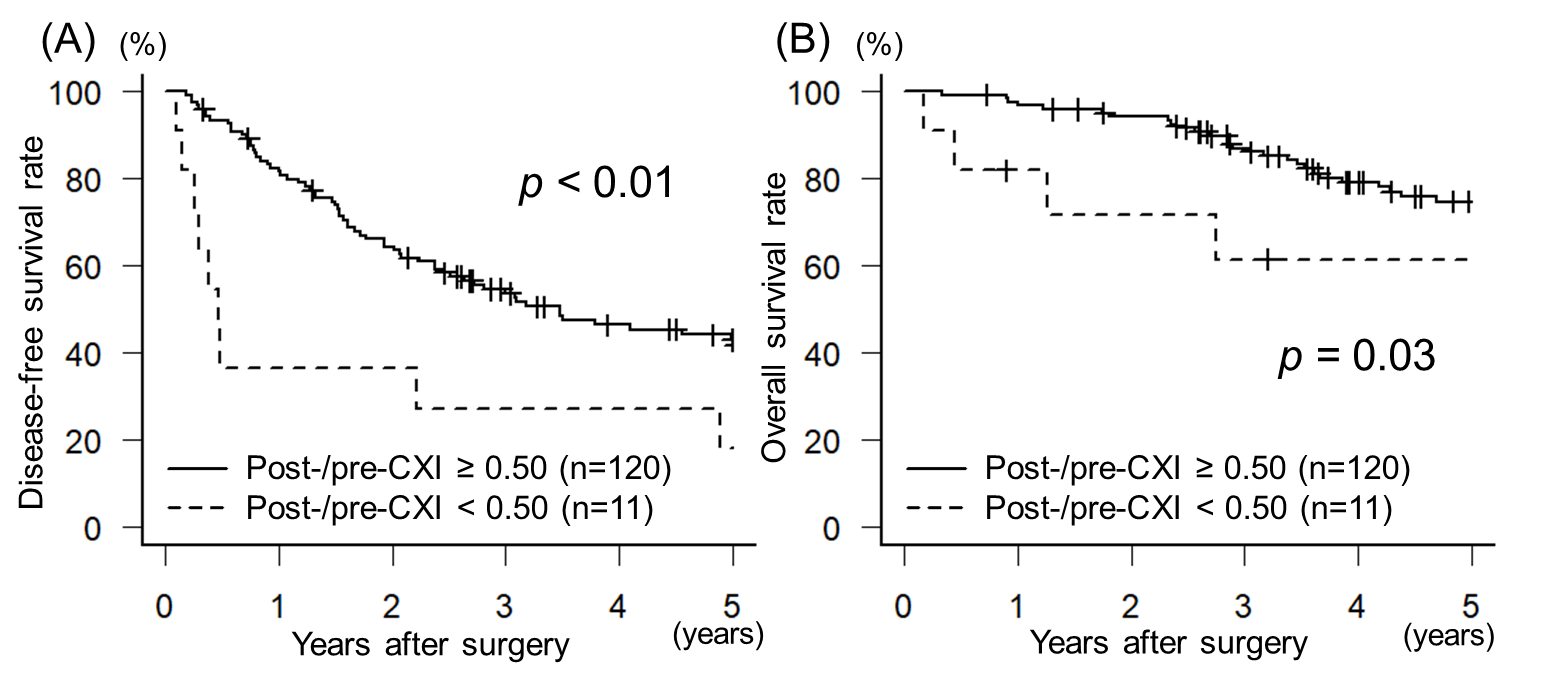


Kaplan-Meier curves of disease-free survival (A) and overall survival (B) after hepatic resection for hepatocellular carcinoma according to the status of the ratios of perioperative change in CXI. Low ratio of perioperative change in CXI was significantly associated with worse disease-free survival (*p*<0.01) and overall survival (*p*=0.03).

**Supplementary Table 1.** Univariate and multivariate analyses of prognostic factors for disease-free survival in patients with hepatocellular carcinoma after hepatic resection: an exploratory analysis using the ratio of perioperative change in cachexia index (CXI).

| Variables | Univariate analysis | |  | Multivariate analysis | |
| --- | --- | --- | --- | --- | --- |
|  | HR (95% CI) | *p*-value |  | HR (95% CI) | *p*-value* |
| Age, ≥ 65 years | 1.19 (0.75-1.87) | 0.46 |  |  | NS |
| Sex, female | 0.70 (0.35-1.40) | 0.31 |  |  | NS |
| HBsAg, positive | 0.43 (0.23-0.80) | <0.01 |  | 0.48 (0.25-0.93) | 0.03 |
| HCV-Ab, positive | 1.06 (0.83-1.37) | 0.64 |  |  | NS |
| Preoperative ICG_R15,_ ≥ 15% | 1.25 (0.81-1.92) | 0.32 |  |  | NS |
| Child-Pugh grade, B | 0.84 (0.34-2.09) | 0.71 |  |  | NS |
| Preoperative serum AFP level, ≥ 20 ng/mL | 1.39 (0.90-2.17) | 0.14 |  |  | NS |
| Preoperative serum PIVKA-II level, ≥ 200 mAU/mL | 1.91 (1.23-2.96) | <0.01 |  | 2.51 (1.50-4.21) | <0.01 |
| Tumor differentiation, poor | 2.06 (1.18-3.60) | 0.01 |  | 2.29 (1.26-4.17) | 0.01 |
| Tumor size, > 5 cm | 1.81 (1.16-2.83) | <0.01 |  |  | NS |
| Tumor number, multiple | 2.17 (1.37-3.44) | <0.01 |  | 3.24 (1.93-5.43) | <0.01 |
| Microvascular invasion, yes | 2.49 (1.47-4.24) | <0.01 |  | 2.66 (1.48-4.79) | <0.01 |
| Type of resection, anatomical | 0.80 (0.51-1.29) | 0.33 |  | 0.42 (0.24-0.72) | <0.01 |
| Operation approach, open | 1.29 (0.62-2.69) | 0.49 |  |  | NS |
| Duration of operation, ≥ 360 min | 1.03 (0.66-1.61) | 0.89 |  |  | NS |
| Intraoperative blood loss, ≥ 1000 g | 1.49 (0.94-2.37) | 0.09 |  |  | NS |
| Intraoperative BTF, yes | 1.76 (1.07-2.89) | 0.03 |  |  | NS |
| Postoperative complication, yes | 1.88 (1.21-2.90) | <0.01 |  | 2.10 (1.29-3.42) | <0.01 |
| Sarcopenia, yes | 1.47 (0.96-2.27) | 0.08 |  |  | NS |
| Preoperative CXI, low | 2.28 (1.32-3.95) | <0.01 |  | 4.80 (2.42-9.53) | <0.01 |
| Postoperative CXI, low^†^ | 1.53 (0.93-2.54) | 0.10 |  |  | NS |
| Ratio of perioperative change in CXI, low | 2.71 (1.40-5.27) | <0.01 |  | 3.65 (1.73-7.68) | <0.01 |

Abbreviations: AFP, alpha-fetoprotein; BTF, blood transfusion; CI, confidence interval; CXI, cachexia index; HBsAg, hepatitis B surface antigen; HCV-Ab, hepatitis C virus antibody; HR, hazard ratio; ICG_R15_, retention rate of indocyanine green at 15 min; PIVKA-II, protein induced by vitamin K absence or antagonist-II; NS, not significant.

^†^ The cut-off value of postoperative CXI for each sex was determined based on the receiver operating characteristic analysis of survival status at 5-year follow-up, which was different from that of preoperative CXI.

* The multivariable Cox regression model initially included age (≥ 65 vs. < 65 years), sex (female vs. male), HBsAg status (positive vs. negative), HCV-Ab status (positive vs. negative), preoperative ICG_R15_ (≥ 15 vs. < 15%), Child-Pugh grade (B vs. A), preoperative serum AFP level (≥ 20 vs. < 20 ng/mL), preoperative serum PIVKA-II level (≥ 200 vs. < 200 mAU/mL), tumor differentiation (poor vs. well or moderate), tumor size (> 5 vs. ≤ 5 cm), number of tumors (multiple vs. solitary), microvascular invasion (yes vs. no), type of resection (anatomical vs. partial), operation approach (open vs. laparoscopic), duration of operation (≥ 360 vs. < 360 min), intraoperative blood loss (≥ 1,000 vs. < 1,000 g), intraoperative BTF (yes vs. no), postoperative complication (yes vs. no), sarcopenia (yes vs. no), preoperative CXI (low vs. high), postoperative CXI (low vs. high), and ratio of perioperative change in CXI (low vs. high). A backward elimination was conducted with a threshold *p* of 0.05 to select variables for the final models.

**Supplementary Table 2**. Univariate and multivariate analyses of prognostic factors for overall survival in patients with hepatocellular carcinoma after hepatic resection: an exploratory analysis using the ratio of perioperative change in cachexia index (CXI).

| Variables | Univariate analysis | |  | Multivariate analysis | |
| --- | --- | --- | --- | --- | --- |
|  | HR (95% CI) | *p*-value |  | HR (95% CI) | *p*-value* |
| Age, ≥ 65 years | 0.94 (0.50-1.78) | 0.86 |  |  | NS |
| Sex, female | 0.69 (0.25-1.94) | 0.48 |  |  | NS |
| HBsAg, positive | 0.39 (0.15-0.99) | 0.047 |  |  | NS |
| HCV-Ab, positive | 1.31 (0.94-1.82) | 0.12 |  |  | NS |
| Preoperative ICG_R15,_ ≥ 15% | 1.96 (1.04-3.69) | 0.04 |  | 2.25 (1.12-4.51) | 0.02 |
| Child-Pugh grade, B | 1.67 (0.59-4.73) | 0.33 |  |  | NS |
| Preoperative serum AFP level, ≥ 20 ng/mL | 1.69 (0.90-3.17) | 0.10 |  |  | NS |
| Preoperative serum PIVKA-II level, ≥ 200 mAU/mL | 1.50 (0.80-2.82) | 0.20 |  |  | NS |
| Tumor differentiation, poor | 2.01 (0.95-4.24) | 0.07 |  | 3.42 (1.54-7.60) | <0.01 |
| Tumor size, > 5 cm | 1.92 (1.02-3.60) | 0.04 |  |  | NS |
| Tumor number, multiple | 1.79 (0.93-3.43) | 0.08 |  |  | NS |
| Microvascular invasion, yes | 2.08 (1.02-4.27) | 0.04 |  |  | NS |
| Type of resection, anatomical | 0.89 (0.46-1.70) | 0.71 |  |  | NS |
| Operation approach, open | 2.05 (0.49-8.52) | 0.32 |  |  | NS |
| Duration of operation, ≥ 360 min | 1.21 (0.63-2.31) | 0.57 |  |  | NS |
| Intraoperative blood loss, ≥ 1000 g | 1.89 (1.00-3.56) | 0.048 |  |  | NS |
| Intraoperative BTF, yes | 2.74 (1.44-5.21) | <0.01 |  | 2.96 (1.47-5.96) | <0.01 |
| Postoperative complication, yes | 2.51 (1.34-4.69) | <0.01 |  | 2.82 (1.45-5.50) | <0.01 |
| Sarcopenia, yes | 2.05 (1.08-3.89) | 0.03 |  |  | NS |
| Preoperative CXI, low | 3.29 (1.63-6.64) | <0.01 |  | 5.11 (2.33-11.21) | <0.01 |
| Postoperative CXI, low^†^ | 2.57 (1.34-4.93) | <0.01 |  |  | NS |
| Ratio of perioperative change in CXI, low | 2.49 (1.04-5.93) | 0.04 |  | 5.21 (2.00-13.53) | <0.01 |

Abbreviations: AFP, alpha-fetoprotein; BTF, blood transfusion; CI, confidence interval; CXI, cachexia index; HBsAg, hepatitis B surface antigen; HCV-Ab, hepatitis C virus antibody; HR, hazard ratio; ICG_R15_, retention rate of indocyanine green at 15 min; PIVKA-II, protein induced by vitamin K absence or antagonist-II; NS, not significant.

^†^ The cut-off value of postoperative CXI for each sex was determined based on the receiver operating characteristic analysis of survival status at 5-year follow-up, which was different from that of preoperative CXI.

* The multivariable Cox regression model initially included age (≥ 65 vs. < 65 years), sex (female vs. male), HBsAg status (positive vs. negative), HCV-Ab status (positive vs. negative), preoperative ICG_R15_ (≥ 15 vs. < 15%), Child-Pugh grade (B vs. A), preoperative serum AFP level (≥ 20 vs. < 20 ng/mL), preoperative serum PIVKA-II level (≥ 200 vs. < 200 mAU/mL), tumor differentiation (poor vs. well or moderate), tumor size (> 5 vs. ≤ 5 cm), number of tumors (multiple vs. solitary), microvascular invasion (yes vs. no), type of resection (anatomical vs. partial), operation approach (open vs. laparoscopic), duration of operation (≥ 360 vs. < 360 min), intraoperative blood loss (≥ 1,000 vs. < 1,000 g), intraoperative BTF (yes vs. no), postoperative complication (yes vs. no), sarcopenia (yes vs. no), preoperative CXI (low vs. high), postoperative CXI (low vs. high), and ratio of perioperative change in CXI (low vs. high). A backward elimination was conducted with a threshold *p* of 0.05 to select variables for the final models.
